# SUCCESSFUL METHODS FOR ELICITING VIEWS OF DIRECT CARE WORKERS REGARDING NEEDS AND PREFERENCES OF HOME CARE CLIENTS

**DOI:** 10.1093/geroni/igad104.3609

**Published:** 2023-12-21

**Authors:** Joan Hyde, Alanna Frost

**Affiliations:** University of Massachusetts, Boston, Massachusetts, United States; University of Massachusetts, Boston, Massachusetts, United States

## Abstract

Over the past decades, as the recruitment and retention of Direct Care Workers (DCWs) has become a critical issue, numerous studies have sought to elicit workers’ views, particularly on job satisfaction, stressors, and intent to stay. This project, which includes a review of those studies, uncovered a wide variety of methods and approaches, which we are evaluating for use in future studies that would capture direct care worker’s insights regarding the needs and preferences of the people for whom they provide support. In particular, this project, in conjunction with a larger one designed to assess dually-eligible care recipients’ experiences with formal home care, explores methodologies best suited to capturing the congruence of DCWs’ views with those of recipients’ needs and preferences. It would test the hypothesis that DCWs’ assessments may supplement recipients’ insights, and may strengthen advocacy to improve home care access, policies and practices. The presentation will offer a systematic review of methods and instruments, including use of validated tools and those with face validity that addresses culturally relevant factors, as well as the use of focused and hypothesis-generating open-ended interviews that are sensitive to the power relationships and voices of those participants in the endeavor. The presenters will offer potentially innovative practices, such as observation, documentation analysis, and the use of Delphi techniques which draw on the knowledge and expertise of DCWs. The presentation will also draw on studies that utilized technology and social media to reach and engage this study population.

